# Prevalence of Cardiovascular Disease (CAD) due to industrial air pollutants in the proximity of Islamabad Industrial Estate (IEI), Pakistan

**DOI:** 10.1371/journal.pone.0300572

**Published:** 2024-07-17

**Authors:** Umer Khayyam, Muhammad Rayan, Iftikhar Hussain Adil

**Affiliations:** 1 Department of Development Studies, School of Social Sciences and Humanities (S^3^H), National University of Sciences and Technology (NUST), Islamabad (ICT), Pakistan; 2 School of Spatial Planning, Chair of Landscape Ecology and Landscape Planning (LLP), Technical University Dortmund, Dortmund, Germany; 3 Department of Economics, School of Social Sciences and Humanities (S^3^H), National University of Sciences and Technology (NUST), Islamabad (ICT), Pakistan; University of Agriculture Faisalabad, PAKISTAN

## Abstract

Contaminated air quality, in lieu of massive industrial pollution, is severely attributing to health anomalies in the proximity of industrial units. Cardiovascular Disease (CAD) is rising around industrial units in the planned capital city of Pakistan, Pakistan. To study self-reported CAD in the proximity of Industrial Estate Islamabad (IEI) by equating two distinct study groups as ‘Band-I’: the residence 0–650 meters and ‘Band-II’ 650–1300 meters radius around the perimeter of IEI. The perimeters were digitized using Google Earth and GIS. Field survey was conducted on deploying 388 (194 in each Band) close-ended (self-administered) questionnaires at the household level, after adjusting the potential confounding variables. The research calculated odds ratios (ORs) of the CAD at 95% CI. The study’s findings of the multiple logistic regression for ORs confirmed a significant increase in CAD problems due to industrial affluents in Band-I than in Band-II which were less severe and less life-threatening. Study confirmed high incidences of high blood pressure and breathing issues (up to 67%), due to accumulation of unhealthy affluents thus leading to heart stroke (Band I = 56.20% and Band II = 60.30%). It is aided by smoking that has increased CAD in Band-I. Societal attributes of knowledge, beliefs, attitudes, and preferences fail to safeguard the local residents amid high concentration of harmful pollutants. As a counter measure the affected respondents engaged in highlighting the issue to the concerned public offices, yet there is a high need on part of the capital government to take mitigative measures to immediately halt the disastrous industrial air emissions to save precious lives.

## 1. Introduction

Urban air quality is deteriorating rapidly because of the surge in demographic and industrial growth [1–4]. This industrial development, further revolutionized with technological boom, though powered the world economies but simultaneously brought a great deal of challenges and harms. One such challenge associated is environmental degradation [5] and affects human health [6, 7]. These advances intend to meet the rising needs of an ever-growing population, thus leading the world societies to scale up industrial processes and operations. It would be naïve to ignore the contribution of industrial processes to the economic growth, poverty reduction, and improving lifestyle fronts. Yet, the harsh reality of environmental degradation and declining human health still paramount the positive impacts [8]. So, with the passing time, the mushroom growth of industrial units undeniably imposing unwanted consequences for the economic benefits as well. The profit of economic growth is costing up to $8.1 trillion, equal to 6.1% of the global GDP [9]. Whereas, at the human-health front, the adverse consequences of the industrial process are linked to most persistent and the most harmful particulate matter (PM_2.5_ and PM_10_) as Nitric oxide, Sulphur Dioxide, Ozone, heavy metals, and Carbon Monoxide (MO)–highly detrimental to public health [10].

The ground situation presents that 80% of the locals are being exposed to ambient air pollution due to industrial emissions. These contaminants in the air quality exceed the World Health Organization’s (WHO) standard, resulting in 4.2 million to 7 million fatalities annually. This means that the inhabitants living near industrial facilities are the highly vulnerable strata of the population [11–14]. These residents encountered both chronic (reduced life expectancy and proliferation in cardiovascular and respiratory disease) and acute (rise in daily fatality and morbidity) health problems due to increased air pollution [15]. Furthermore, releasing inimical pollutants into the air can adversely affect the Air Quality [16], leading to various gastrointestinal disorders (Table 1).

**Table 1:** Major industrial units and their respective emissions.

Industries	Pollutants
Thermal Power Plant	NO_2_, N_2_O, SO_2_
Steel Industries	Smoke, Particulates, CO_2,_ Fluoride
Petroleum Refineries	Smoke, Particulates, SO_2_
Metal Smelters	Smoke, Particulates, NO_2_, N_2_O, SO_2_
Fertilizer Plant	NO_2_, N_2_O, SO_2_
Soap and detergent Plants	Particulates, Odour
Marble industries	Particulates, Odour, SO

*Source*: [26]

Outdoor atmospheric pollution stands high in low-middle income countries, where South-Asia comprises of some most polluted cities globally. Accordingly, only 0.4% South-Asian cities are currently meeting WHO PM_2.5_ standard [17]–accountable for 12–17% of all CVD fatalities [18]. In South-Asian, amid growth through industrial boom, Pakistan stands 3^rd^ most polluted country [17], besides amongst countries where air pollution his emerging as another disaster [19], hence posing serious threat to the country’s public health [20]. City which was known for its pure environment is also not save from the atmospheric pollutants.

Unlike other cities of Pakistan, Islamabad is the only planned city of the country having dedicated sectors e.g., industrial sectors (I-sectors) and the residential areas (sectors other than I-sectors). Only in this city, the bifurcation between houses and the industrial units was planned to keep a safe distance between humans and the industrial affluents. Yet, the buffer between (exclusively) the residential areas and industrial area/estates is blown-up with time as the area in the industrial (I) sectors was sold for residential/housing purposes that has immediately resulted in building of houses near to industrial sectors [21]. As a result, the air quality of Islamabad (the capital city) got exposed to fast phase degradation amid Industrial Estate Islamabad (IEI), having direct health impacts on the residents living close to industrial production units. The industrial units in the city remain as the primary source of injecting contaminated gases and metal particulates into the open air [22] (Table 1). The total suspended particulate concentrations of in Islamabad are twice higher as other nearby (industrial) zones [23]. It is determined that the pollutants (such as NO_2_, N_2_O, SO_2_, Smoke, Particulates, CO_2_, Fluoride and odour) originating from IEI (namely; mainly ghee, marble, steel, flour, chemical, paint, soap, pharmaceutical and pigments) are the key drivers that not only significantly contribute to environmental problems in Islamabad [24], but alarmingly causing serious health issues amongst the nearby inhabitants [6, 25]. Amongst other health diseases, development of cardiovascular diseases in the nearby residents are very high. It is, therefore, IEI in the capital (planned) city remains important to be studied amid rising CAD diseases.

### 1.1. CAD & the case of IEI

According to rough estimates, currently there are 1200 industries operating in Islamabad, whereas around 500 industries are operational in sectors I-9 and I-10 [27]. These industries are situated dangerously close (within 1300 m.) to residential areas, hence remain detrimental to public health. The health issue in the form of cardiovascular diseases (amongst other e.g., gastrointestinal, and digestive tract infections) are allegedly found dominant amongst inhabitants residing around industrial units [28–30]. High rate of health degradation in the vicinity is linked to industrial contaminants (such as nitrogen oxides, carbon monoxide etc.) infused into the open atmosphere causing human health to suffer [10, 28, 31–33]. This rising rate of human health degradation in Islamabad is the reflection of City’s air pollutants crossing the National Environmental Quality Standards (NEQS) limits as well as the exceeding the WHO 24-hour exposure limit of 15μg/m3 [34]. Yet, Islamabad’s high air concentration of PM_2.5_ stands 20 times higher: equal to 173μg/m3 [17]. This high concentration of PM_2.5_ is persistent, which is further aided by the city’s local environment, especially during summers when the air temperature remains avg. 24°C aiding to transportation of industrial effluents and facilitate by rise in the mean air temperature by 0.8°C [25]. Further, the wind speed (1.33 m/s) and the wind direction (from North, Northeast, Northwest and Southwest and intermittently from South and West) with no air advection [35, 36] helps pollutants to disperse in all directions [37, 38]. Thus, the prevalence and concentration of industrial air pollutants during (pre-)summer are declared very high.

Globally the societies have already moved to cleaner and sustainable production models. These models under the fourth industrial revolution (IR 4.0) are linked to the use of smart and efficient technologies to not only control but also reduce harmful industrial emissions to safeguard natural environment as well the lives. Due to lack of access and affordability to cleaner technology, financial as well human capital the global South countries are lagging. This is because, the poor countries like Pakistan, are confronted with brunt of industrial pollution as well as paramounting nuisance of outcomes of their development. These issues, coupled with energy intensive fossil fuel consumption and lack of transformative attempts towards cleaner production methods are resulting into GHG emissions. In addition, industrial units in the country and in IEI failed to deploy pollution control measures (e.g., effluent treatment systems). It is coupled with low environmental regulations’ compliance (e.g., compliance with National Environment Quality Standards-NEQS). Slow shift to sustainable and green methods, and rising industrial pollution are wreaking havoc on the health of exposed communities.

### 1.2. Aim and research questions

The aim of this study to find out the prevalence of CAD due to industrial air pollution in the proximity of IEI Pakistan.

1. What is prevalence level of CAD amongst the residents of IEI?
2. What types of preventive measures considered by the affected population to protect themselves and to which extend they are effective?
3. What is the level of government response to tackle mounting CAD in the capital city?

## 2. Research methodology

### 2.1. Study area and industries

**Fig 1:**
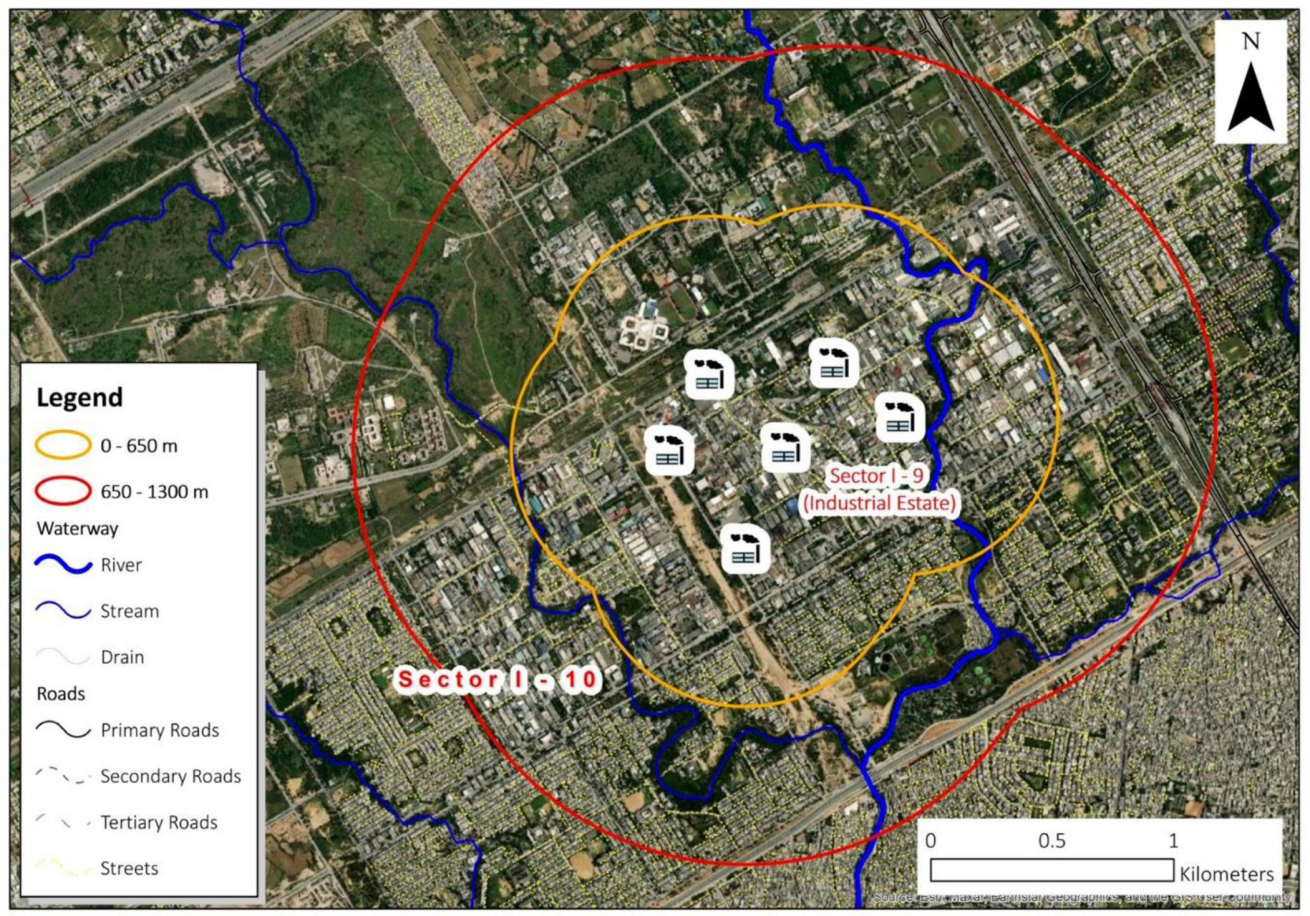
GIS area map of the study area—IEI (Source: Authors).

The study area Islamabad Industrial Estate (IEI) is in the Industrial zone/sector of the capital city of Islamabad. Islamabad is situated at 33.7214813°N (latitude) and 73.0432892°E (longitude), elevating around 600m. above the sea level [39]. The best plan city in the country, Islamabad is divided into different sectors, thereby separating the industrial sectors from the residential sectors. In the master plan of Islamabad, the residential and industrial sectors are separated through a buffer zone. Industrial Estate Islamabad (IEI) is dispersed over and area of 625 acres. It constitutes in-total 475 industrial units (260 in I-9, and 215 in I-10) [40, 41]. These industries are located close to each other, thereby developing one consolidated area surrounded by the residential houses–all within the perimeter of 1300 meters around industries (Fig 1, if accepted).

### 2.2. Study design & area

This research is conducted to study the prevalence of cardiovascular diseases nearby local industries due to industrial air pollution by deploying cross-sectional method–which facilitates to deploy an observational assessment to not only check the level of contact with the emission but also the consequences on human health in the study population. This type of approach facilitates predicting and assessing (time specific) exposure and the outcome on human health than doing any likely or retrospective study as follow-ups [42].

This study has taken the ‘level of exposure’ and ‘the health impacts in the form of CAD’ as dependent (study) variable, whereas residential/study area or distance from the industrial units (outer boundary) in I-9 and I-10 sectors (Fig 1), along with gender, age, education level, income, smoking habits and occupational exposure.

### 2.3. Band selection & exposure geocoding

To position of industries (IEI) as well location of the residential area is certified through their exact addresses by using grid references as referred by Pakistan Bureau of Statistics (Islamabad). Geographical Information System (ArcGIS version 10.9, ESRIInc.) Google^TM^ Earth are used for the extraction of perimeters of IEI to set the distance bands. Here, two distance bands Band-I (0–650 meters) and Band-II (650–1300 meters) are considered, referring to the IEI perimeters. The distance bands are validated by Environmental Protection Agency (EPA Pakistan), besides being supported through the relevant literature (Table 2). It is to study the prevalence of air pollutants and their harmful health impacts up to 1300 meters from the source point. These bands are not synonym of pollutants’ movement (or homogenous distribution of pollutants), yet the movement of the pollutants is reported homogenously in all directions [6].

**Table 2:** Distance bands I&II.

Distance Band	Description
BAND-I 0–650 meters	Pakistan-Environmental Protection Agency (Pak.-EPA) has ratified the distance bands. It is also confirmed that the industrial pollutants as released from industries in IEI lay at their maximum level [6, 43]
BAND-II 650–1300 meters	The industrial air pollutants are detected in this range/band at an unbearable level which are seldom found beyond the 1300m limit, according to Pak.-EPA [6, 44]

### 2.4. Sampling and data collection

**Fig 2:**
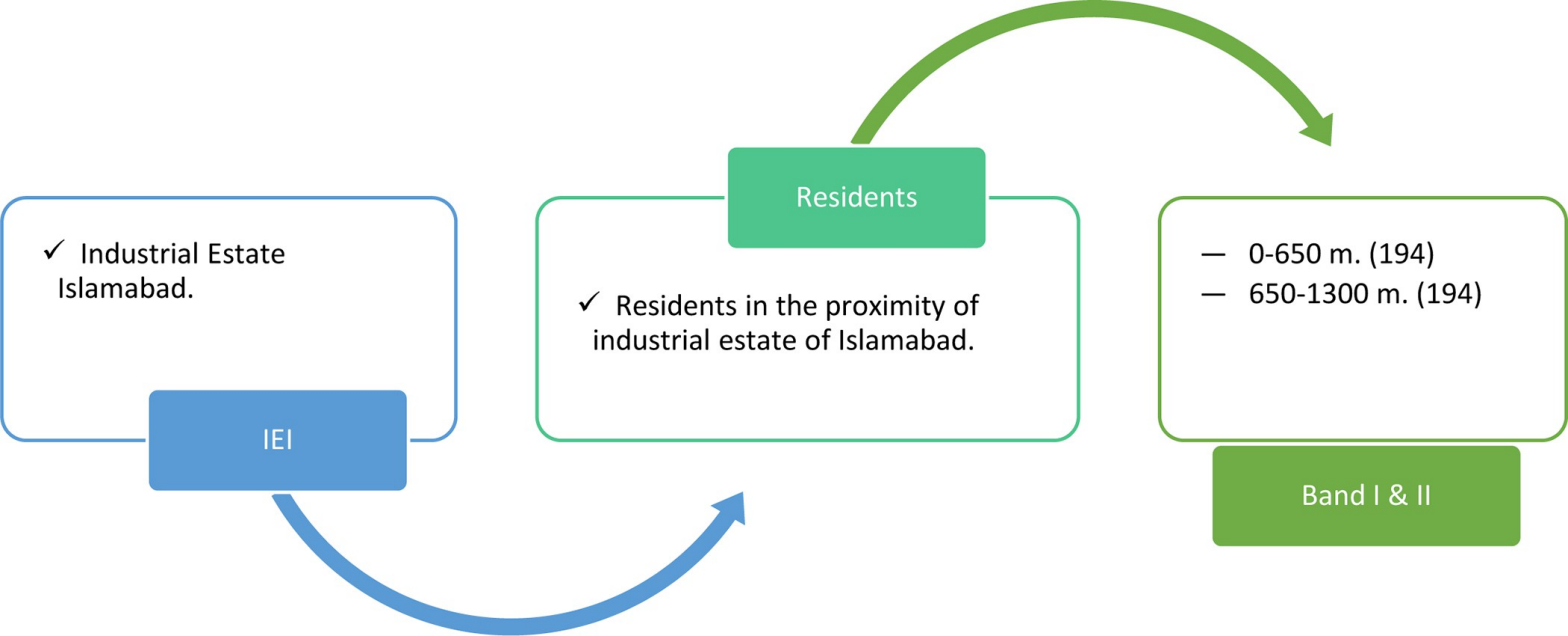
Flow diagram.

Primary data is collected through field survey by deploying 388 (194 in each sector) structured questionnaires which are self-administered at the Household (HH) level. The sample size was calculated based on procedure suggested by Cochran (1963) with 95% CI ±5 MoE. This facilitates to generalize the results to other areas with similar characteristics and faced with similar problem due to air pollution. The close-ended structures questionnaire was divided in to two different sections: socio-demographics and then questions related to CAD and its leading health symptoms. Also, the questions related to societal preventive measures (e.g., protest, complaints logged, contracting Pak.-EPA etc.) (Fig 2)

**Fig 3:**
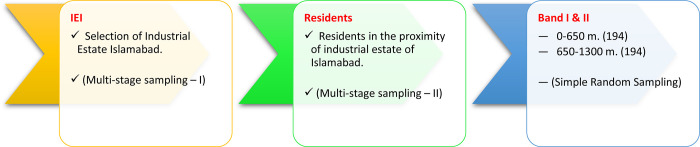
Sampling design: *Multi-stage sampling*.

For quality data collection through field survey, multistage purposive sampling technique is used, as frequently deployed in studying health impacts as self-reported [45]. At the first stage: Islamabad as study area is selected. At the second stage; IEI is selected through purposive sampling as there is no other industrial area in the city. Thirdly; the samples (HHs) are selected on simple random sampling, equally (194 each) in both the bands (Fig 3). Primary data collection is done when industries were fully operational–which confirms saturation open air with industrial pollutants [46]. Further, during times when wind speed is high and temperature is rising the concentration of air pollutants remains also high [47]. It ensured that data collected is of high quality as it is collected at peak industrial emission time, in the most polluted industrial sectors of Islamabad, from the most affected native residents that are residing around the industrial zones. Also, the section of the study variable (CAD) remains the most important component to be studied, which is not studied sofar in this area.

### 2.5. Empirical modelling & data analysis

This research study has used both descriptive statistics and inferential statistics. The earlier is deployed to abridge the data distribution, whereas the latter is used for data analysis to identify associations, congregation and the degree of variance in the study/dependent variable as of independent variable. The associations between self-reported CAD problems (apropos distance from the source point) after adjusting other confounders like, gender, age, income, education, smoking status, and occupational exposure are computation via adjusted odds ratios. For this purpose, SPSS Statistics (V.25) is used for multiple logistic regression to study the linkage of dependent variable, on adjusted odds to address confounding effects in human-health studies (Table 3).

**Table 3:** Variables with descriptions.

Variable	Description	Source
**Independent Variable**		
Distance from industries	0-650m, 650-1300m	[43]
***Potential Confounder***		
Gender	Male, Female	[43]
Age (years)	<30, 30–60, >60	[48]
Education	<Matric, UG, PG&PhD	[49]
Income (PKR)	<50k, 50k-10k, >10k	[43]
Smoking	Never, Sometimes, Smoker	[50]
Occupational exposure	Exposed, Not exposed	[51]
**Dependent/Study Variable**	Cardiovascular Diseases (CVD): Heat Attack/stroke (high amongst children and elderly people	[34, 52, 53]

### 2.6. [Potential] confounders

For robustness of the results and to avoid any negative effects on the study’s results, potential confounders are adjusted stepwise in the model. It is to check effect of the point of interest (distance) on the residents’ health. Further, the confounders are adjusted in the model to check the prevalence of the disease, so a stepwise selection is used in both the model building as well as analysis. The exposure of interest (distance from the source point) remains in the model; however, other variables are entered one-by-one into each model. It is pertinent to indicate that the statistical models do support epidemiological research on enabling (every individualistic) outcome’s prediction as conditional on independent variable or by gauging the effects of risk factors adjusted as covariates. Therefore, it is concluded that that the effect estimates, besides the fact that usual methods used for confidence interval are also valid [54]. Lastly, as the literature suggest a significant and direct relation between exposure to air pollution and risk perception measurment [55], validate the accurate sample selection and model building of the present study.

## 3. Results and discussions

### 3.1. Demographic characteristics

The cross-tabulation test (chi-square analysis) was used to study demographic characteristics of study subject of participants from Band I and Band II. According to the test, majority of the respondent from both bands were between the age group of 30–60 years. The percentage for this age category for Band I was 52.1% and it was 52.6% for Band II. Majority of the respondents from both bands were male, having a percentage of 67% for Band I and 63.4% for Band II. Major chunk of the respondents from both bands were either in/completed post graduate degree or were in/completed PhD. The percentage under this variable for Band I and Band II was 70.1% and 74.7%, respectively. For smoking status, participants were given three options i.e., smoker, smokes sometimes, and those who never smoked. Majority of the participants from both bands were smokers, having a percentage of 45.9 for Band I and 56.9 for Band II. For Household HH income, of all the three categories, majority of the participants from both bands had income between 50k-10k. The percentage for this category was 52.6 for both bands. The variable “occupational exposure to dust/fumes” was found significant (*p* value 0.04). Majority of the participants were not exposed to dust and fumes at their occupation. The percentage for Band I was 76.3 whereas it was 84.5 for Band II (Table 4).

**Table 4:** Demographic characteristics of study subject of both the distance bands.

Demographic Characteristics	Distance Bands		*P*-Value
	*Band I*	*Band II*	
**Age (%)**			
<30	43.8%	42.3%	0.86
30–60	52.1%	52.6%	
60>	4.1%	5.2%	
**Gender (%)**			
Male	67%	63.4%	0.45
Female	33%	36.6%	
**Education**			
None-Matric	6.7%	5.7%	0.59
College-UG	23.2%	19.6%	
PG-PhD	70.1%	74.7%	
**Smoking status (%)**			
Smoker	45.9%	56.9%	0.97
Sometimes	28.9%	28.4%	
Never	25.3%	24.7%	
**Household income**			
<50k	19.6%	13.9%	0.23
50k-10k	52.6%	52.6%	
>10k	27.8%	33.5%	
**Occupational Exposure to Dust/Fumes (%)**			
Exposed	23.7%	15.5%	0.04*
Non-Exposed	76.3%	84.5%	

* P < 0.05 shows significant relation

### 3.2. Association of health problems with the proximity of IEI

**Fig 4:**
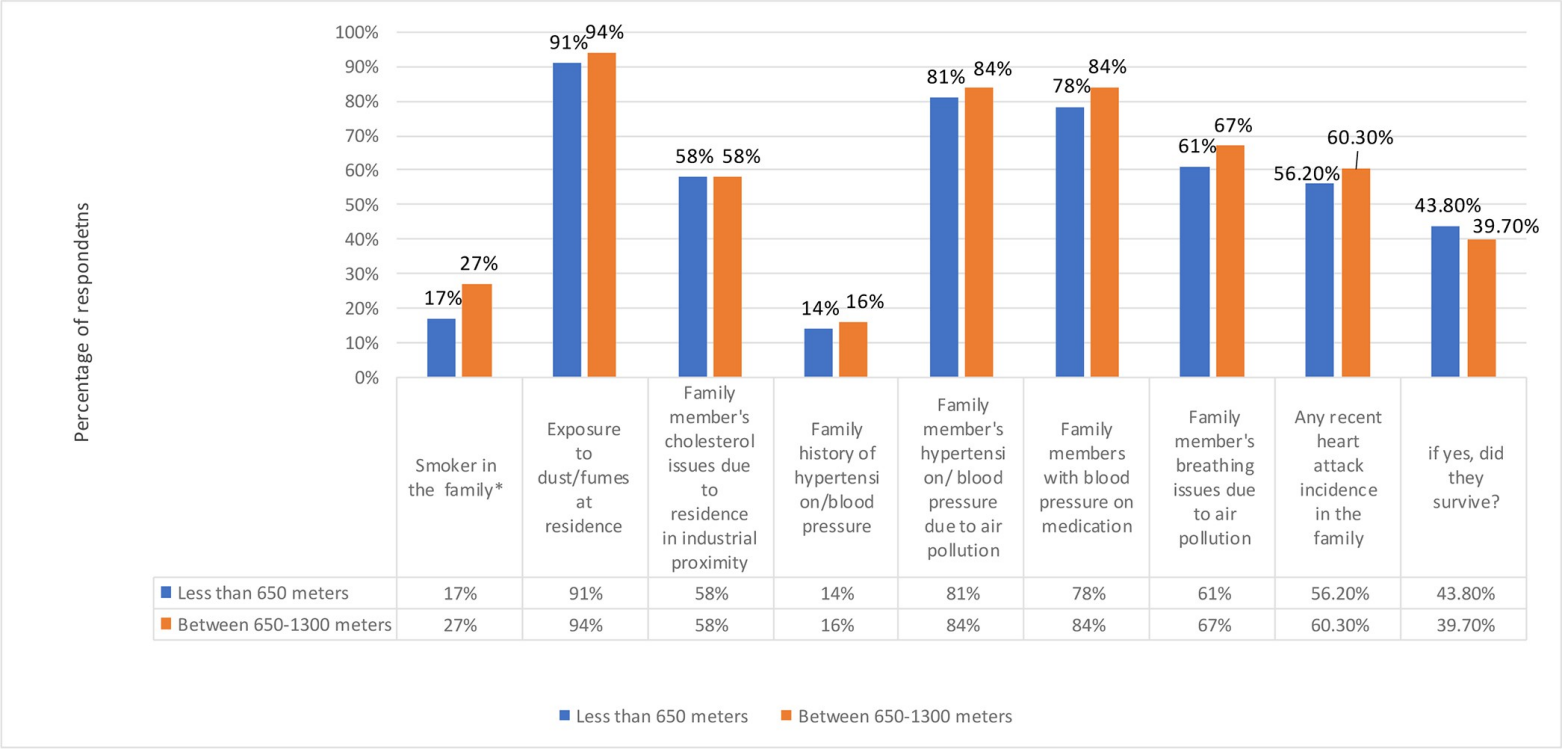
Percentages of health issues with respect to distance band. * P value less than 0.05 shows significance.

In the present study, HHs from Band I had more smokers than HHs from Band II (17% VS 27%). The variable was found to be significant and had a *p* value less than 0.05. HHs from Band I were more exposed to dust/fumes at residence than HHs from Band II (91% VS 94%). Equal percentage (58%) was found for “Family member’s cholesterol issues due to residence in industrial proximity” for HHs of both bands. For “Family member’s hypertension/ blood pressure due to air pollution” majority of the HHs from Band I agreed that air pollution triggered the hypertension/ blood pressure in their family members. The percentage for both bands was 81% and 84%, respectively. More HHs from Band II were having blood pressure issues (14% VS 16%). For “Family members with blood pressure on medication”, majority of HHs from Band I were on medications than HHs from Band II (78% VS 84%). HHs from Band II were more in the view that family member’s breathing issues are due to air pollution (61% VS 67%). Majority of the HHs from Band II had recent heart attack incidence in their family in comparison to HHs from Band II (56.20% VS 60.30%), whereas majority of the participants from HHs of Band I survived these incidences (43.80% VS 39.70%) (Fig 4).

### 3.3. Potential confounders

The study included certain confounders, which have the potential to affect the accurate model building. These included Age, Gender Education, Smoking status, Household income and Occupational Exposure to Dust/Fumes. These were adjusted with the help of multiple logistic regression to establish a strong relationship between the independent and dependent variable. For calculation of the Adjusted Odds Ratios (AOR) the independent variable and all the confounders were taken simultaneously, whereas the dependent variable was taken one by one as shown in Table 5. For logistic regression, the reference category for gender was male, whereas for Income, PKR 10k and more was chosen as the reference category. For Education PG, PhD was taken as the reference category, whereas for age, less than 30 years was taken as the reference category. For smoking status, smoker was selected as the reference category whereas for occupational exposure to dust/fumes, non-exposed was taken as the reference category. Results of the AORs from multiple logistic regression analysis are shown in Table 5 below.

**Table 5:** Adjusted ORs and the association between distance bands and health issues.

**Distance Bands**	**Smoker in the family**			**Exposure to dust/fumes at residence**			**Family member’s cholesterol issues due to residence in industrial proximity**		
	AOR	(95% CI)^a^	P Value*	AOR	(95% CI)^a^	P Value*	AOR	(95% CI)^a^	P Value*
	1.74	(1.05,2.88)	0.03*	1.87	(0.82,4.29)	0.13	1.05	(0.68,1.60)	0.82
	**Family history of hypertension/blood pressure**			**Family member’s hypertension/blood pressure due to air pollution**			**Family members with blood pressure on medication**		
	AOR	(95% CI)^a^	P Value*	AOR	(95% CI)^a^	P Value*	AOR	(95% CI)^a^	P Value*
	1.15	(0.64,2.05)	0.63	1.18	(0.68,2.02)	0.54	1.55	(0.89,2.68)	0.11
	**Family member’s breathing issues due to air pollution**			**Any recent heart attack incidence in the family**			**if yes, did they survive?**		
	AOR	(95% CI)^a^	P Value*	AOR	(95% CI)^a^	P Value*	AOR	(95% CI)^a^	P Value*
	1.21	(0.79,1.86)	0.36	1.21	(0.79,1.83)	0.36	0.82	(0.54,1.25)	0.37

^a^ Adjusted Odds Ratio (95% confidence interval) for living in less than 650 meters adjusted for Gender: Male (reference), Income: Pkr 10k and more (reference category), Distance: less than 650 meters (reference), Smoking status: smoker (reference), Education: PG, PhD (reference category), Age: <30 (reference category), Occupational exposure: non-exposed(reference)

* P value less than 0.05

After adjusting the odds, only smoke in the family significantly increased in Band I by 1.74 in comparison to band II (having a *p* value of 0.03). However, all other variables were found to be insignificant (*p* value more than 0.05).

### 3.4. Societal preventive measures vs. health issues

**Fig 5:**
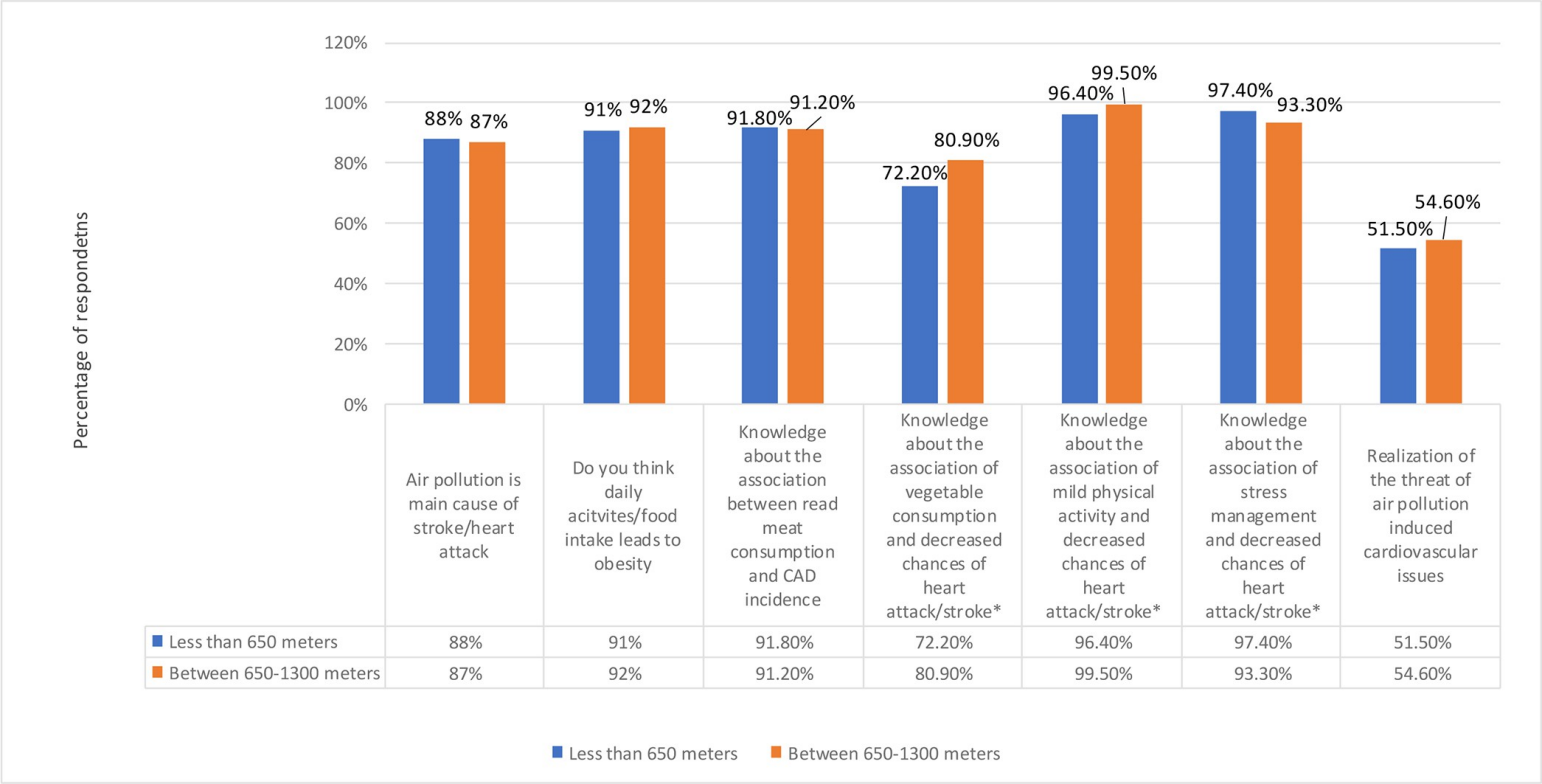
Preventive measures and reduced health issues w.r.t distance bands. * P value less than 0.05 shows significance.

In the present study, almost equal percentages of HHs from Band I and Band II thought that air pollution is main cause of stroke/heart attack (88% VS 87%). Similar trend was observed for the next variable. HHs from both bands reported that they think daily activities/food intake leads to obesity (91% VS 92%). About the knowledge about the association between read meat consumption and CAD incidence, HHs from both bands agreed that they knew about the relationship (91.80% VS 91.20%). About knowledge about the association of vegetable consumption and decreased chances of heart attack/stroke, majority of the HHs from Band II agreed that they knew about the relationship i.e., 72.20% VS 80.90 (*p<* 0.05). Similar trend was reported for knowledge about the association of mild physical activity and decreased chances of heart attack/stroke, having percentages of (96.40% Vs 99.50%). This variable also had a *p* value less than 0.05. More HHs from Band I agreed that they had the knowledge about the association of stress management and decreased chances of heart attack/stroke (97.40% VS 93.30%). The variable was found to be significant (*p* value less than 0.05). For “realization of the threat of air pollution induced cardiovascular issues”, HHs from Band II, agreed more (51.50% VS 54.60%) (Fig 5).

**Fig 6:**
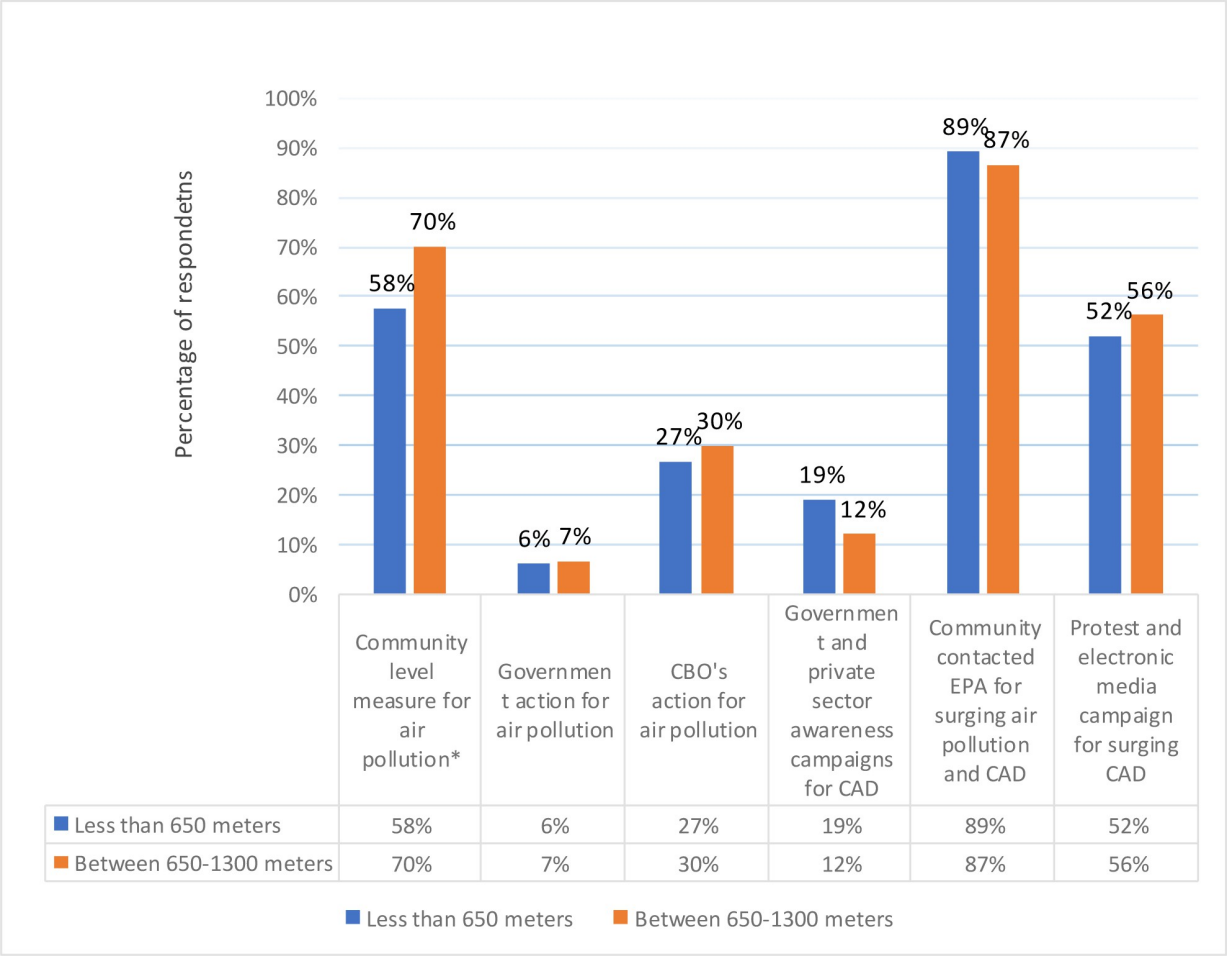
Distance from the source and adoption of preventive measures. * P value less than 0.05 shows significance.

After adjusting the odd, the odds of having the knowledge about the association of vegetable consumption and decreased chances of heart attack/stroke was more for HHs of band I by 1.74 than for HHs of band II. The variable had a significance value of 0.03. The odds of having the knowledge about the association of mild physical activity and decreased chances of heart attack/stroke among the HHs of band I was 13.8 in comparison with the HHs of Band II (*p* value = 0.01). However, unlike the previous variables, the odds of having the knowledge about the association of stress management and decreased chances of heart attack/stroke, were more for HHs of Band II (*p* value = 0.04). All other variables were found to be statistically insignificant *p* value greater than 0.05 (Fig 6 and Table 6).

**Table 6:** Adjusted ORs: Preventive measures and reduced health issues.

**Distance Bands**	**Air pollution is the main cause of stroke/heart attack**			**Do you think daily activities/food intake leads to obesity**			**Knowledge about the association between read meat consumption and CAD incidence**		
	AOR	(95% CI)^a^	P Value*	AOR	(95% CI)^a^	P Value*	AOR	(95% CI)^a^	P Value*
	.906	(0.47,1.72)	0.76	1.09	(0.50,2.37)	0.81	0.99	(0.47,2.09)	0.99
	**Knowledge about the association of mild physical activity and decreased chances of heart attack/stroke**			**Knowledge about the association of stress management and decreased chances of heart attack/stroke**			**Realization of the threat of air pollution induced cardiovascular issues**		
	AOR	(95% CI)^a^	P Value*	AOR	(95% CI)^a^	P Value*	AOR	(95% CI)^a^	P Value*
	13.8	(1.55,124)	0.01*	0.32	(0.10,0.97)	0.04*	1.10	(0.72,1.68)	0.64

^a^ Adjusted Odds Ratio (95% confidence interval) for living in less than 650 meters adjusted for Gender: Male (reference), Income: PKR 10k and more (reference category), Distance: less than 650 meters (reference), Smoking status: smoker (reference), Education: PG, PhD (reference category), Age: <30 (reference category), Occupational exposure: non-exposed(reference).

* P value less than 0.05 shows significance

### 3.5. Socio-demographic characteristics vs. knowledge about preventive measures

HHs from Band II were more in the view that community level measures are taken for air pollution (58% VS 70%). The variable was found to be statistically significant and had a *p* value less than 0.05. Similar trend was observed for the variable “government action for air pollution”. The variable had percentages of 6% VS 7% for Band I and Band II, respectively. HHs from Band II believed that CBO’s action against air pollution were taken (Band II-30%). More HHs from Band I believed that there were government and private sector awareness campaigns for CAD (19% VS 12%). Almost equal percentages of HHs from both bands believed that community contacted EPA for surging air pollution and CAD (89% VS 87%). More HHs from band II reported that there were protests and electronic media campaign for surging CAD (52% VS 56%) (Table 7).

**Table 7:** Socio-demographic characteristics and knowledge about preventive measures.

Demographic Characteristics	*Knowledge* about Preventive Measures	*P-val*.
**Age (%)**		
<30	Air pollution is the main cause of stroke/ heart attack	0.00*
<30	Do you think daily activities/ food intake leads to obesity?	0.00*
<30	Knowledge about the association of vegetable consumption and decreased chances of heart attack/stroke	0.04*
<30	Realization of the threat of air pollution induced cardiovascular issues	0.00*
>60	Knowledge about the association between read meat consumption and CAD incidence	0.001*
30–60 and >60	Knowledge about the association of mild physical activity and decreased chances of heart attack/stroke	0.004*
**Income (%)**		
50k-10k	Knowledge about the association of stress management and decreased chances of heart attack/stroke	0.002*
**Occupational Dust/Fumes**		
Not Exposed	Realization of the threat of air pollution induced cardiovascular issues	0.03*

* *P* < 0.05 shows significant relation

After adjusting the potential confounders, only the odds of community level measure for air pollution increased significantly (*p* value = 0.01) by 1.68 in Band I in comparison to Band II. Adjusted odd ratios for all other variables were found to be statistically insignificant (*p* value more than 0.05) (Table 8).

**Table 8:** Adjusted odds ratios for collective measures for CAD and air pollution.

**Distance Bands**	**Community level measure for air pollution**			**Government action for air pollution**			**CBO’s action for air pollution**		
	AOR	(95% CI)^a^	P Value*	AOR	(95% CI)^a^	P Value*	AOR	(95% CI)^a^	P Value*
	1.68	(1.08,2.60)	0.01*	1.23	(0.52,2.86)	0.63	1.16	(0.73,1.83)	0.51
	**Government and private sector awareness campaigns for CAD**			**Community contacted EPA for surging air pollution and CAD**			**Protest and electronic media campaign for surging CAD**		
	AOR	(95% CI)^a^	P Value*	AOR	(95% CI)^a^	P Value*	AOR	(95% CI)^a^	P Value*
	0.66	(0.36,1.21)	0.18	0.76	(0.40,1.43)	0.40	1.14	(0.74,1.74)	0.54

^a^ Adjusted Odds Ratio (95% confidence interval) for living in less than 650 meters adjusted for Gender: Male (reference), Income: PKR 10k and more (reference category), Distance: less than 650 meters (reference), Smoking status: smoker (reference), Education: PG, PhD (reference category), Age: <30 (reference category), Occupational exposure: non-exposed(reference)

* P value less than 0.05 shows significance

## 4. Discussion & conclusion

This research contributes to finding out the prevalence of cardiovascular diseases due to industrial air pollution injected by the IEI, affecting the nearby population of I-9 and I-10 sectors in the capital city of Pakistan; Islamabad. This paper contributes to the existing literature on the topic by showing how increasing industrial air pollution creates health problems (like CAD) for the inhabitants, especially in the post COVID-19 scenario. This empirical study has outlined an explicit quantitative research methodology of self-reporting to dissect the prevalence of air pollutants and their harmful health impacts on the local dwellers; stretched to two distinct study bands as validated by EPA-Pakistan and Bano and Khayyam, 2021 (e.g., short distance up to 650 m as ’Band-I’, and long-distance (650–1300) as ’Band-II’, framed around IEI.

It is found that the local community is facing blood pressure and breathing issues (up to 67%), coupled with incidents of heart attack (Band I = 56.20% and Band II = 60.30%). This coincides with the scientific evidences that exposure to industrial pollutants (like being emitted from IEI) is directly linked to increased incidence of CAD [56]. Further, it is aided that family (active and passive) smoking history increased by 1.74 in Band-I. All other confounding variables were found to be insignificant, *p<* 0.05). It is further reported from Band I and Band II (88% and 87% respectively) that air pollution remains the main cause of stroke/heart attack amongst residents, this remains apparent due to industrial air pollutants [57]. It is now evident that air quality in Pakistan’s main cities like Islamabad is consistently exceeds the set guidelines [58, 59].

The results of the regression model represent that only (smoke in the family) variable was found significant in a Band- I, compared to band II (p-value<0.05). While studying ‘knowledge, beliefs, attitudes, and preferences of the HHs from both bands w.r.t preventive measures’ this study has established that awareness of daily activities/food intake to minimize heart attack, an association between read meat consumption and CAD incidence (upto 91%), role of vegetable consumption to decrease stroke (up to 80%), physical activity and stress management to safeguard their lives from CAD etc. (all variables found significant, p-value<0.05)–despite of their realization and healthy activities the bulk of industrial affluents in the open air in the area is taking away their health and lives. Yet, other studies also hint at small evidences of the coronary heart diseases like in China [60]. Which links to counter measures against the uncontrolled air pollution and rising CAD around production units. It is found that community level measures (once taken) to highlight the issue to the concerned public offices, can help. But, measures like, contacting EPA to report the upsurge of air pollution and CAD (up to 89%) and protests through electronic media campaigns (upto 56%) led to limited, if non-existent. It has paving-way to burn more fossil-fuels and pollute the air by the profit seeking industrialists.

It can be concluded that the lack protection policy, weak laws, non-compliance/enforcement to monitor toxic air pollutants and tackle mounting CAD effects in the urban areas like Islamabad are missing. So much so, the implementation of plan to shift industrial units away from the residential areas (to I-17) is yet to many more years to materialize. So, to support the green growth and a clean environment, we need proactive and pragmatic long-term planning policies and strategies implementation for green/clean air action reform programs to prevent all chronic and acute (like CAD) health problems. Furthermore, promoting community stewardship is also essential in policymaking, awareness, and public sensitization to ensure clean air, especially in the non-collaborative and unilateralism environment; that Pakistan possesses.

### 4.1. Policy implications

1. Restoration of buffer zone between residential areas and IEI on immediate basis and the same for future industrial projects at the national level.
2. Monitoring of toxic air pollutants including carcinogens, PM_2.5_, ozone precursors and heavy metals and more detailed studies on occupational and residential cohorts, biomonitoring, epidemiological investigations etc. to systematically monitor the issues in the capital city, besides the enactment of National Environment Policy and Pakistan Clean Air Program (2005) to ensure clean air via improvement of standards of stationary and mobile sources.
3. Public sensitization, awareness, and education to adapt to efficient protective measures for personal protection against the air toxins.

## Supporting information

S1 File (DOCX)
