# Supplementary material for: Prevalence of Cardiovascular Disease (CAD) due to industrial air pollutants in the proximity of Islamabad Industrial Estate (IEI), Pakistan
Source: PLoS One. 2024 Jul 17;19(7):e0300572. doi: 10.1371/journal.pone.0300572 (PMC11253970; doi:10.1371/journal.pone.0300572)
Supplement: S1 File — (DOCX) [file pone.0300572.s001.docx]

**Prevalence of Cardiovascular Disease (CAD) due to Industrial Air Pollutants in the Proximity of Islamabad Industrial Estate (IEI), Pakistan.**

- Cardiovascular Disease (CAD) due to industrial air pollution is studied in the ‘planned’ city of Islamabad, Pakistan.
- The vicinity around Industrial Estate Islamabad (IEI) was divided into two distinct groups: ‘Band-I’: 0-650 and ‘Band-II’ 650–1300 meters radius around the perimeter of IEI to study the prevalence of CAD according to distance.
- Study confirmed high incidences of blood pressure and breathing issues due to the accumulation of unhealthy affluents, thus leading to heart stroke.
- Societal attributes of knowledge, beliefs, attitudes, and preferences were found limited to safeguard the local residents amid high concentration of harmful pollutants.
- High need of critical technology is required in the post-COVID 19 times as nations are alarmingly rising industrial affluents amid economic growth.
- The study calls for strict compliance of separation of residents from industrial areas around the world to safeguard human lives.
